# The association between serum brain-derived neurotrophic factor and a cluster of cardiovascular risk factors in adolescents: The CHAMPS-study DK

**DOI:** 10.1371/journal.pone.0186384

**Published:** 2017-10-13

**Authors:** Natascha Holbæk Pedersen, Jakob Tarp, Lars Bo Andersen, Anne Kær Gejl, Tao Huang, Lone Peijs, Anna Bugge

**Affiliations:** 1 Centre of Research in Childhood Health, Department of Sports Science and Clinical Biomechanics, University of Southern Denmark, Odense, Denmark; 2 Department of Teacher Education and Sport, Western Norway University of Applied Sciences, Campus Sogndal, Norway; 3 Norwegian School of Sport Sciences, Department of Sports Medicine, Oslo, Norway; 4 Department of Physical Education, Shanghai Jiao Tong University, Shanghai, China; 5 The Centre for Physical Activity Research, Department of Infectious Diseases, Rigshospitalet, University of Copenhagen, Denmark; Chiba Daigaku, JAPAN

## Abstract

**Background and objective:**

Cardiovascular disease and type 2 diabetes pose a global health burden. Therefore, clarifying the pathology of these risk factors is essential. Previous studies have found positive and negative associations between one or more cardiovascular risk factors and brain-derived neurotrophic factor (BDNF) probably due to diverse methodological approaches when analysing peripheral BDNF levels. Moreover, only a few studies have been performed in youth populations. Consequently, the main objective of this study was to examine the association between serum BDNF and a composite z-score consisting of six cardiovascular risk factors. A secondary aim was to examine the associations between serum BDNF and each of the six risk factors.

**Methods:**

Four hundred and forty-seven apparently healthy adolescents between 11–17 years of age participated in this cross-sectional study. Cardiorespiratory fitness (CRF), anthropometrics, pubertal status, blood pressure (BP), serum BDNF, high-density lipoprotein cholesterol (HDL-C), triglyceride (TG), blood glucose and insulin were measured. Information about alcohol consumption and socio-economic status was collected via questionnaires. Associations were modelled using linear regression analysis.

**Results:**

Serum BDNF was positively associated with the composite z-score in the total study sample (standardized beta coefficient (std.β) = 0.10, P = 0.037). In males, serum BDNF was positively associated with the composite z-score (Std. β = 0.14, P = 0.034) and HOMA-IR (Std. β = 0.19, P = 0.004), and negatively associated with CRF (Std. β = -0.15, P = 0.026). In females, BDNF was positively associated with TG (Std. β = 0.14, P = 0.030) and negatively associated with waist circumference (WC) (Std. β = -0.16, P = 0.012).

**Conclusion:**

Serum BDNF was positively associated with a composite z-score of cardiovascular risk factors. This association seems to be mainly driven by the association between TG, HOMA-IR and serum BDNF, and particularly for males. Further longitudinal research is warranted to determine the temporal relationship between BDNF and cardiovascular risk factors.

## Introduction

Lifestyle-related diseases such as cardiovascular disease (CVD) and type 2 diabetes mellitus (T2DM) are enormous global health burdens. In 2012, these two major non-communicable diseases caused a total of 19 million deaths worldwide [1]. Risk factors for CVD include hyperglycaemia, dyslipidaemia, hypertension and abdominal obesity [2]. These risk factors appear more often together than by chance alone. In adults this clustering of cardiovascular risk factors is often referred to as the metabolic syndrome [2]. In children and adolescents, however, no universal definition of the metabolic syndrome exists [3]. Furthermore, cut-offs are arbitrarily chosen as children and adolescents do not fit the adult definition of the metabolic syndrome [4]. Therefore, some authors have proposed a continuous composite z-score of risk factors as a better method to identify children and adolescents with a higher cardiovascular risk than their peers [3, 5]. By the utilization of this method clustering of cardiovascular risk factors has been identified in children as young as nine years old [4].

Clarifying the underlying pathology causing the simultaneous rise in the cardiovascular risk factors is essential for targeting prevention and disease control. Recently, brain-derived neurotrophic factor (BDNF) was identified to play a potential role in this amplification [6, 7]. BDNF is a neurotrophin protein crucial for the differentiation, maturation and survival of neurons. Together with the other neurotrophin proteins (NGF, NT3 and NT4/5), BDNF modulates both electrical properties and the structural organization of the synapse by binding to the unselective low-affinity necrosis factor receptor P75^NTR^ and its high-affinity receptor tropomyosin-related kinase B (TrkB) [8, 9]. High levels of both BDNF and TrkB have been identified in the hypothalamus, which is a brain region important in appetite- and glucose regulation, weight control, and energy homeostasis [7, 9]. A recent study found that the deletion of TrkB (destroying the putative causal chain) in the hypothalamus in mice resulted in increased body weight, adiposity and impaired glucose homeostasis [10]. It has been hypothesized that BDNF has metabotrophic potential and may have and important role in cardiometabolic homeostasis maintenance [11]

Accordingly, a number of studies have examined the association between peripheral BDNF and one or more of the cardiovascular risk factors in humans, however, results point in different directions. Examining the geriatric population, Golden *et al*. [12] found that circulating plasma BDNF levels were positively associated with body mass index (BMI), body fat mass, blood pressure (BP), total cholesterol (TC) and low-density lipoprotein cholesterol (LDL-C). This concurs with a study by Levinger *et al*. [13], who found positive associations between plasma BDNF levels and triglyceride (TG), plasma glucose, plasma insulin and abdominal fat in middle-aged healthy individuals. Likewise, Bus *et al*. [14] found positive associations between serum BDNF and the presence of metabolic syndrome and coronary heart disease in adults. However, when analyses were adjusted for age the associations were lost. When examining single risk factors, Bus *et al*. [14] only found a positive association between BDNF and TG. In contrast, a study of patients with angina pectoris found that the associations between plasma BDNF and TG, LDL-C and the presence of T2DM were inverse [15]. Therefore, the adult literature is conflicting. El-Gharbawy *et al*. [16] found that serum BDNF levels were significantly lower in extremely overweight children and adolescents compared to normal weight controls. To our knowledge, no studies have investigated the association between BDNF and BP, TG, cholesterol and insulin resistance in a healthy population of adolescents. Thus, research is warranted to clarify these relationships.

The main objective of this study was to examine the association between serum BDNF and a composite z-score consisting of six cardiovascular risk factors; inverse of high-density lipoprotein (HDL-C), TG, BP, insulin resistance (HOMA-IR), waist circumference (WC) and inverse of cardiorespiratory fitness (CRF). As a secondary objective, the associations between serum BDNF and each of the six risk factors were examined.

## Materials and methods

### The CHAMPS-study DK

The CHAMPS-study DK was initiated in 2008. It was conducted as a quasi-experimental longitudinal study evaluating the effects of a tripling of physical education (PE) from 1.5 to 4.5 hours per week in six primary schools in the municipality of Svendborg, Denmark. Four matched schools served as controls. The primary aim of the CHAMPS-study DK was to assess the health effects of the PE intervention [17]. The present study solely included results from the most recent follow-up conducted in 2015. Thus, this study is a cross-sectional analysis. The extended methods of the CHAMPS-study DK are described elsewhere [18]. Only measurements and methods relevant for this investigation are presented below.

### Recruitment and participation

In 2015, 1457 adolescents attending 6^th^ to 10^th^ grade were invited to participate in a follow-up of the CHAMPS-study DK. In total, 745 subjects provided written informed consent obtained from a parent or a legal guardian. Of these subjects, 705 of the 1457 adolescents (48% of invited) participated in the testing. Subjects with metabolic dysfunctions (diabetes, hyperglycaemia etc.) were excluded (5 subjects). In total, 447 (31% of invited) had complete data and were included for analyses in this study. The Regional scientific Ethical Committee (Region of Southern Denmark) approved the 2015 supplemental protocol (Project number: S-20140105).

### Data collection

All measurements were collected at the schools in 2015 from February till April. Standardized and detailed testing protocols were designed to ensure data quality. Experienced research assistants gathered data on anthropometrics, pubertal status, BP and CRF. Trained biomedical laboratory technicians drew all blood samples.

### Questionnaires

Two questionnaires were completed; one by a parent/legal guardian and one by the student. In the parental questionnaire information was gathered about the mother’s level of education, which was used as indicator of socioeconomic status (SES). SES was coded into four groups (1. high school or less, 2.vocational education, 3. short tertiary education or 4. bachelor level and above).

In the student questionnaire information was gathered about alcohol consumption, which was coded in five groups (1. I never drink alcohol, 2. I drink alcohol less than once a month, 3. I drink alcohol once a month, 4. I drink alcohol a couple of times a month or 5. I drink alcohol a couple of times a week). The latter two categories were collapsed due to a low number in each category leaving four categories.

### Anthropometrics and pubertal status

Anthropometric measurements included body mass, height and waist circumference (WC). Body mass was measured to the nearest 0.1 kg (Tanita BWB-800S, Tanita Corporation, Tokyo, Japan). Height was measured to the nearest 0.5 cm (SECA 214, Seca Corporation, Hanover, MD, USA). WC was measured at the level of the umbilicus after a light expiration. Two measurements of body height and WC were conducted and yet another one if the measurements differed with more than 1 cm. Pubertal status was assessed using the Tanner pubertal stages self-assessment questionnaires [19]. Subjects were asked to indicate which of five pubertal stages (pictures) matched themselves in regards to the males’ pubic hair development and the females’ breast development. The questionnaires were completed in a private, separated room. Since only one subject belonged to pubertal stage 1, the adolescent was excluded from analyses.

### Cardiorespiratory fitness (CRF)

After a five minutes warm-up and thorough instructions subjects completed an intermittent running test using the Andersen test protocol [20]. Covered running distance during the test was reported. Research assistants verbally encouraged all subjects.

### Blood pressure

Systolic BP (BPsys) was measured in an isolated room with quiet surroundings. BPsys was measured after five minutes of rest on the left arm using an automated oscillometric blood pressure monitor (Omron 705IT, Omron, Kyoto, Japan). The measurement was repeated a minimum of five times with two minutes intervals and the mean of the three final measures with readings within five mmHg was used.

### Blood samples

Blood samples were collected at the schools after an overnight fast to analyse serum BDNF, TC, HDL-C, TG, glucose and insulin. Blood samples used for serum BDNF analyses were put directly on ice. Subsequently, all blood samples were transported to the laboratory. The blood samples were centrifuged with 1000g for 15 minutes at room temperature. Time between blood drawing and centrifugation was <4 hours (time was noted). Blood samples were stored 4–6 months at -80°C. Sandwich ELISA kits (Quantikine, R&D Systems, Minneapolis, MN, USA) were used to measure levels of serum BDNF. Samples were analyzed in duplicates, and the mean concentrations were used in the statistical analyses. Analyses were performed at the Centre for Physical Activity Research, Department of Infectious Diseases, Rigshospitalet, University of Copenhagen, Denmark.

TC, TG, glucose and HDL-C were analysed by quantitative determination using enzymatic, colorimetric method on Roche/Hitachi cobas c system (Roche, Mannheim, Germany). Insulin was analysed using solid phase enzyme labeled chemiluminescent immunometric assay. Insulin resistance was estimated by the homeostasis model assessment, HOMA-IR; blood glucose (mmol/L) x insulin (μU/mL) divided by 22.5 [21].

### Statistical analyses

Pearson’s chi^2^ test (categorical data), unpaired t-test (normally distributed data) and Wilcoxon rank sum test (non-normally distributed data) were used for gender comparisons in the study population (Table 1). HDL-C, TG, BPsys, HOMA-IR, WC and CRF were the risk factors included in the composite z-score. Before generating the composite z-score, variables (risk factors) that did not follow a normal distribution were log-transformed (insulin, TG and WC). Furthermore, HDL-C and CRF were multiplied by -1 (only in the composite score) as these variables are inversely related to cardiovascular health. Risk factors were standardized by regressing age, gender and pubertal status on the respective risk factors and calculating the standardized residuals. These residuals are by definition independent of the predictor variable with a mean of zero and a standard deviation of one. WC and BPsys were further standardized by height, and all blood measurements (except BDNF) were standardized by weekday as preliminary analyses revealed an association between weekday and TG and insulin. This observation has been reported elsewhere [22]. The final composite z-score was a mean of the six included risk factors (z-scores). The composite score was converted to a standardized value. A higher value represents a less favourable risk profile.

**Table 1:** Characteristics of the study population stratified by gender.

	Females	Males
Number	214	233
Age (years)	14.09 (1.29)	14.44 (1.26)*
Body height (cm)	164.28 (7.10)	170.00 (11.15)*
Body weight (kg)	53.31 (8.87)	55.98 (12.19)*
Body-mass index (kg/m^2^)	19.68 (2.51)	19.16 (2.42)*
Waist circumference (cm) ^a^	70.00 (66.00–74.75)	71.50 (67.75–76.50)*
Serum BDNF (ng/ml)	26.98 (6.05)	27.01 (6.34)
Insulin resistance (HOMA-IR) ^a^	1.48 (1.14–1.98)	1.20 (0.88–1.68)*
Total cholesterol (mg/dl)	155.24 (24.94)	143.67 (23.49)*
High-density lipoprotein cholesterol (mg/dl)	56.93 (13.02)	54.08 (13.25)*
Systolic blood pressure (mm Hg)	105.59 (8.24)	109.58 (8.89)*
Triglyceride (mg/dl) ^a^	64.00 (51.00–80.00)	58.00 (46.00–76.00)*
Cardiorespiratory fitness (m)	1046.79 (95.47)	1146.75 (104.41)*
Standardized composite z-score	0.00 (1.03)	0.00 (0.97)
Tanner stage (%) 1 2 3 4 5	0% 4% 42% 44% 10%	0%* 9% 26% 45% 19%
Socio-economic status (%) High school or less Vocational education Short tertiary education Bachelor level and above	7% 26% 11% 56%	6% 31% 14% 49%
Alcohol consumption (%) I drink a couple of times a month I drink once a month I drink less than once a month I never drink	11% 8% 9% 72%	16% 4% 13% 67%

Characteristics presented as percentage, mean (SD) or median (interquartile range).

^a^: Variables expressed as median (interquartile range) due to non-normality.

*: Significant difference between gender (P<0.05).

The association between serum BDNF and the composite z-score was examined using linear regression analysis. The analyses were adjusted for age, gender (unless stratified), pubertal status, SES and alcohol consumption. Further inclusion of a “random-effect” at the school-class level to model any within-cluster correlation revealed that only 5.4% of the variation (ICC = 0.054) was explained at this level, hence we modelled the association using the more parsimonious ordinary least squares model. Regression analyses examining the association between serum BDNF and single risk factors were conducted using the same statistical approach and adjustments as above. In a separate model we included a multiplicative interaction term between gender and BDNF. Prior to analyses we specified a secondary gender-stratified model because platelet BDNF levels may vary across the female menstrual cycle [23] and we only include the gender x BDNF interaction term to formally test for difference in slopes (Wald test) between the genders. All models were examined for assumptions of normality and homoscedasticity of residuals and for linearity between independent and dependent variables. All analyses were completed using Stata 14.0 (StataCorp, College Station, Texas, USA). We used α <0.05 (two-sided) as the level of significance.

## Results

### Characteristics of the study population

Four hundred and forty-seven participants completed all measurements included in this study. The participants in the analytic sample had significantly lower BMI and HOMA-IR and significantly higher CRF and height compared to the sample of subjects who had missing data (S1 Table). Moreover, the two samples were significantly different with respect to alcohol consumption and SES.

Characteristics of the study population are presented in Table 1. Males and females were significantly different with respect to pubertal status, age, body weight, body height, BMI, WC, HOMA-IR, TC, HDL-C sysBP, TG and CRF (p<0.05). However, no significant differences were found between genders in BDNF levels, SES and alcohol consumption (p>0.05).

### Associations between serum BDNF and a composite z-score

In the total sample, BDNF levels were significantly and positively associated with the composite z-score (P = 0.037). When the analyses were dichotomized by sex, BDNF levels were positively associated with the composite z-score (P = 0.034) for males, but the association was not significant for females (P = 0.548) (Table 2). Gender and serum BDNF did not interact in predicting the composite z-score (P = 0.204).

**Table 2:** Associations of serum BDNF (ng/ml) with composite z-score and single cardiovascular risk factors.

	Total			Males			Females		
	β (95% CI)	p-value	Std. β	β (95% CI)	p-value	Std. β	β (95% CI)	p-value	Std. β
Standardized composite z-score	0.60 (0.04;1.17)	0.037*	0.10	0.95 (0.07; 1.82)	0.034*	0.14	0.23 (-0.52; 0.97)	0.548	0.04
Insulin resistance (HOMA-IR)	0.93 (0.37; 1.49)	0.001*	0.15	1.19 (0.39; 1.99)	0.004*	0.19	0.64 (-0.17; 1.45)	0.122	0.10
High-density lipoprotein cholesterol	-0.15 (-0.72; 0.41)	0.597	-0.02	0.02 (-0.81; 0.84)	0.968	<0.00	-0.23 (-1.00; 0.54)	0.555	-0.04
Blood pressure	0.16 (-0.40; 0.73)	0.570	0.03	-0.12 (-0.96; 0.72)	0.778	-0.02	0.52 (-0.24; 1.28)	0.179	0.09
Triglyceride	0.83 (0.27; 1.40)	0.004*	0.13	0.75 (-0.14; 1.64)	0.097	0.11	0.80 (0.08; 1.52)	0.030*	0.14
Waist circumference	-0.33 (-0.89; 0.23)	0.252	-0.05	0.24 (-0.68; 1.15)	0.609	0.03	-0.91 (-1.61; -0.20)	0.012*	-0.16
Cardiorespiratory fitness	-0.29 (-0.86; 0.27)	0.304	-0.05	-1.00 (-1.87; -0.12)	0.026*	-0.15	0.46 (-0.42; 1.33)	0.302	0.07

Regression analyses adjusted for age, gender (unless stratified), pubertal status, SES and alcohol consumption. The standardized composite z-score includes: insulin resistance, triglyceride, blood pressure, waist circumference, inverse of high-density lipoprotein cholesterol and inverse of cardiorespiratory fitness. Note that a composite z-score expresses a high cardiovascular risk as HDL-C and CRF have been multiplied by -1 (in the composite z-score only). Single risk factors are presented as z-scores standardized by age, gender (unless stratified) and pubertal status. Systolic BP and WC were adjusted for height. All non-BDNF blood measurements are adjusted for weekday.

*and bold values = Significant association.

### Associations between serum BDNF and single risk factors

In the total sample, serum BDNF levels were positively associated with HOMA-IR (P = 0.001) and TG (P = 0.004). The same positive association was evident in males between BDNF and HOMA-IR (P = 0.004). Also BDNF levels were negatively associated with CRF in males (P = 0.026). In females, serum BDNF levels were positively associated with TG (P = 0.030) and negatively with WC (P = 0.012) (Table 2).

## Discussion

The main finding in this study is that serum BDNF in apparently healthy 11 to 17 year old adolescents is positively associated with a composite z-score consisting of cardiovascular risk factors. Analyses examining the associations between serum BDNF and single risk factors revealed that especially TG and HOMA-IR seem to contribute to the positive association between BDNF and the composite z-score.

The gender x BDNF interaction term did not reach statistical significance when modelling the composite score as outcome. Previous studies have reported significant differences in BDNF levels between men and women. These differences may be related to estrogen in the females, as studies have found that BDNF levels are associated with estrogen levels throughout the menstrual cycle [24]. Lommatzsch *et al*. [23] found a significant difference in platelet BDNF levels (which are closely related to serum BDNF levels) between women in the first and the last half of the menstrual cycle. Therefore, our choice of a stratification in the analyses was based on an *a priory* expectation of different BDNF levels between the genders. It was seen that the association between BDNF and the composite z-score was driven mainly by the significant association found in males, and the effect size was about four times larger compared to females. These observations should be interpreted in the light of the weakened precision of our estimates when performing stratified analyses and we notice that the sex-specific confidence intervals are wide and overlap. The assumed fluctuating BDNF levels in females (due to BDNF’s association with estrogen levels) may have concealed or blurred a potential association between BDNF and the composite z-score in females. We did not inquire details about the menstruation cycle of participants in our study due to privacy concerns. This may be a limitation of our study.

The data revealed an inverse relationship between BDNF and WC in females. To the best of our knowledge, only one study has examined the association between BDNF and obesity in children and adolescents [16]. El-Gharbawy et al. [16] found that serum BDNF levels were significantly lower in overweight children and adolescents compared to the normal weight controls, which supports the negative association found between BDNF and WC in females in the present study. Based on these observations, measures of body fatness in children and adolescents seem to relate differently to BDNF compared to the other cardiovascular risk factors. However, WC related to the other cardiovascular risk factors as expected (S2 Table). Thus, we are unable to explain the inverse association between serum BDNF and WC.

The rationale behind examining the association between BDNF and a composite z-score of cardiovascular risk factors was to examine whether BDNF levels relate to overall cardiovascular health. However, the significant association observed between BDNF and the composite z-score seems to be driven mainly by the association between BDNF and TG and HOMA-IR, respectively. The positive association found between BDNF and TG has previously been reported in adults and in the geriatric populations [12, 14, 25, 26]. To our knowledge, this is the first study reporting the positive association between BDNF and TG in adolescents. Insulin resistance (HOMA-IR) is strongly associated to coronary heart disease in adults, and it has been found that HOMA-IR predicts clustering of cardiovascular risk factors in children and adolescents [27]. A large body of evidence, both in animals and humans, supports the association between serum BDNF and insulin resistance and glucose metabolism. However, the direction of the association differs between studies. Krabbe *et al*. [28] and Fujimani *et al*. [29] found that low BDNF levels in plasma and serum, respectively, were associated with impaired glucose metabolism and T2DM. Additionally, Karczewska-Kupczewska *et al*. [30] found that serum BDNF levels in non-obese women with low insulin sensitivity were decreased compared to women with high insulin sensitivity. On the other hand, Boyuk *et al*. [26] and Suwa *et al*. [31] found increased serum BDNF levels in T2DM patients compared to healthy controls, which concurs with the findings in the present study. Chaldakov [11] has previously found that circulating BDNF levels are decreased in subjects with advanced metabolic syndrome, whereas BDNF levels are elevated in subjects in the early stages of metabolic disturbances. If BDNF levels change concurrently with the development of metabolic disturbances, this could be a possible explanation for the conflicting results in the field.

### Methodological challenges in BDNF analysis

In recent years numerous methodological challenges regarding BDNF analyses have been highlighted and discussed [32]. Since CNS levels of BDNF are difficult to measure in humans, peripheral levels of BDNF are often used as a proxy. Peripheral BDNF levels can be measured in serum, plasma, whole blood or platelets. However, it is uncertain which method is the best reflection of central BDNF levels. Moreover, levels of serum BDNF are not proportionally related to plasma or whole blood BDNF levels and vice versa [32]. Thus, studies using different measures of peripheral BDNF levels are not directly comparable. Plasma contains anti-coagulates, whereas serum is prepared after coagulation. Some studies have shown that part of the BDNF stored in platelets is released during the clotting process, which causes BDNF concentrations in serum to increase over time[33]. Therefore, serum BDNF level is sensitive to clotting time and storage duration [34, 35]. Animal models have shown that high BDNF levels in the hypothalamus seem to have a positive effect on metabolic health, for example on weight control and glucose regulation [10]. However, in the present study high serum BDNF levels seem to be associated with a higher cardiovascular risk. One explanation for the observed positive associations in our study could be an increased platelet activation, which previously has been observed in pathological conditions (e.g. elevated fasting glucose, endothelia dysfunction and hypertension) [36, 37], and this may cause an increased platelet BDNF release in serum. Possibly, peripheral plasma BDNF levels analyzed in platelet-poor plasma would display associations with CVD risk more similar to that of brain BDNF level. However, more studies are needed to clarify this.

The blood samples in the present study were analysed for serum BDNF using ELISA kits. This equipment does not distinguish between pro-BDNF and mature BDNF, and these two BDNF variants may lead to different biological responses [32]. The mature BDNF protein, which binds to its specific TrkB receptor, seems to promote cell survival and maturation. Pro-BDNF, on the other hand, binds to the p75^NTR^ receptor, which also seems to promote neuronal death and retraction of dendritic spines [38]. The proportions of mature BDNF and pro-BDNF in cells that express TrkB and P75^NTR^ seem to have the ability to determine the balance between cell death and cell survival [9]. In pathological conditions, for example in atherosclerosis and diabetes, vascular cells seem to express the pro-BDNF receptor p75^NTR^ promoting cell apoptosis and inhibiting angiogenesis [39], which may be another explanation for the high BDNF levels observed in subjects with a high cardiovascular risk. Therefore, a method that distinguishes between mature BDNF and pro-BDNF could have been relevant in this area of research, as the direction (positive/negative) and significance of the association between BDNF and cardiovascular risk factors may depend on the variant of BDNF.

### Strengths and limitations

The strength of the present study is the inclusion of a large sample of adolescents. A limited amount of research has been conducted in this population in this area and most with smaller sample sizes. Moreover, the large test battery has provided a range of available information making it possible to adjust for potential confounding factors. Furthermore, the use of a continuous composite z-score provides a more informative picture of overall cardiovascular risk compared to a binary assessment of each cardiovascular risk factor [3, 40].

A limitation in the present study is the cross-sectional design making it impossible to conclude on causality. Based on these data, it is not possible to conclude whether the high serum BDNF levels are a cause, consequence, or bi-product of an increased cardiovascular risk. Moreover, numerous studies have previously concluded that high central BDNF levels are supportive of cardiovascular health, brain health etc. The inverse findings in the present study in peripheral serum BDNF may indicate that serum BDNF levels do not reflect central BDNF levels.

Previous studies have found significant associations between serum BDNF and depression, and BDNF may even play an important role in the association between depression and CVD [41, 42]. Unfortunately, we did not collect data on depression, which is an additional limitation in this study.

Another limitation in the present study is the external validity, since the analytic sample differed significantly from the sample of subjects with missing data (S1 Table). Moreover, this study population is, according to the biological characteristics, a healthy cohort of young people for which reason the observed association between serum BDNF and a composite z-score (and single risk factors) may be different in other adolescents. Further research is warranted to verify our results in other cohorts, and also to investigate the possible biological differences in measuring BDNF in serum, plasma or whole blood, and how this affect the association to cardiovascular risk factors.

## Conclusion

Based on data presented in this study, peripheral serum BDNF seem to be positively associated with a composite z-score consisting of six cardiovascular risk factors. This association is mainly driven by the association between serum BDNF and TG and HOMA-IR, respectively, particularly for males. Further, preferable longitudinal research, is needed to determine the temporal relationship between BDNF and cardiovascular risk factors.

## Supporting information

S1 Table Differences between analytic sample and participants with missing data. Characteristics presented as percentages, mean (SD) or median (interquartile range). ^a^: Variables expressed as median (interquartile range) due to non-normality. *: Significant difference between samples.
(DOCX)

S2 Table Partial correlation coefficients between cardiovascular risk factors and the composite z-score. All variables are represented as z-scores adjusted for age, gender and pubertal status. SysBP and WC were adjusted for height. All non-BDNF blood measurements were adjusted for weekday. *and bold values = Significant correlation.
(DOCX)
