# Supplementary material for: The association between serum brain-derived neurotrophic factor and a cluster of cardiovascular risk factors in adolescents: The CHAMPS-study DK
Source: PLoS One. 2017 Oct 13;12(10):e0186384. doi: 10.1371/journal.pone.0186384 (PMC5640247; doi:10.1371/journal.pone.0186384)
Supplement: S1 Table — Characteristics presented as percentages, mean (SD) or median (interquartile range). a: Variables expressed as median (interquartile range) due to non-normality. *: Significant difference between samples. (DOCX) [file pone.0186384.s001.docx]

**S1 Table:** Differences between analytic sample and participants with missing data

|  | Analytic sample | Participants with missing data | Difference  (P-value) |
| --- | --- | --- | --- |
| Number  Age (years)  Height (cm)  Weight (kg)  Body-mass index (kg/m^2^)  Waist circumference (cm) ^a^  Serum BDNF (ng/ml)  Insulin resistance (HOMA-IR) ^a^  Total cholesterol (mg/dl)  High-density lipoprotein (mg/dl)  Systolic blood pressure (mm Hg)  Triglyceride (mg/dl) ^a^  Cardiorespiratory fitness (m)  Tanner stage (%)  1  2  3  4  5  Socio-economic status (%)  High school or less  Vocational education  Short tertiary education  Bachelor level and above  Alcohol consumption (%)  I drink a couple of times a month  I drink once a month  I drink less than once a month  I never drink | 447  14.27 (1.29)  167.25 (9.85)  54.70 (10.80)  19.41 (2.48)  70.50 (67.00-75.50)  27.00 (6.19)  1.33 (1.00-1.82)  149.21 (24.85)  55.45 (13.21)  107.67 (8.81)  61.00 (48.00-79.00)  1098.89 (111.90)  0 %  7 %  34 %  45 %  15 %  6 %  29 %  13 %  52 %  14 %  6 %  11 %  69 % | 258 or <  14.13 (1.25) (n=258)  165.46 (9.11) (n=257)  54.74 (11.43) (n=254)  19.84 (3.05) (n=254)  71.50 (66.00-76.25) (n=257)  26.92 (5.86) (n=167)  1.58 (1.09-2.49) (n=176)  152.46 (26.65) (n=176)  56.45 (14.59) (n=176)  107.66 (8.66) (n=247)  63.00 (52.00-82.50) (n=176)  1070.54 (107.22) (n=163)    2 % (n=255)  8 %  32 %  47 %  11 %    13 % (n= 176)  32 %  9 %  46 %    19 % (n= 248)  7 %  6 %  69 % | -  0.161  0.017*  0.965  0.043*  0.423  0.883  0.000*  0.151  0.407  0.988  0.108  0.005*  0.050  0.027*    0.034* |

Characteristics presented as percentages, mean (SD) or median (interquartile range). ^a^ : Variables expressed as median (interquartile range) due to non-normality. *: Significant difference between samples.
