# Supplementary material for: The association between serum brain-derived neurotrophic factor and a cluster of cardiovascular risk factors in adolescents: The CHAMPS-study DK
Source: PLoS One. 2017 Oct 13;12(10):e0186384. doi: 10.1371/journal.pone.0186384 (PMC5640247; doi:10.1371/journal.pone.0186384)
Supplement: S2 Table — All variables are represented as z-scores adjusted for age, gender and pubertal status. SysBP and WC were adjusted for height. All non-BDNF blood measurements were adjusted for weekday. *and bold values = Significant correlation. (DOCX) [file pone.0186384.s002.docx]

**S2 Table:** Partial correlation coefficients between cardiovascular risk factors and the composite z-score

|  | Std. composite z-score | HOMA-IR | TG | WC | sysBP | CRF | HDL-C |
| --- | --- | --- | --- | --- | --- | --- | --- |
| Standardized composite z-score  Insulin resistance (HOMA-IR)  Triglyceride (TG)  Waist circumference (WC)  Systolic blood pressure (sysBP)  Cardiorespiratory fitness (CRF)  High-density lipoprotein (HDL-C) | 1.000  0.633  **<0.000***  0.594  **<0.000***  0.617  **<0.000***  0.425  **<0.000***  -0.583  **<0.000***  -0.553  **<0.000*** | 1.000  0.309  **<0.000***  0.299  **<0.000***  0.081  0.086  -0.320  **<0.000***  -0.144  **<0.000*** | 1.000  0.096  **0.044***  0.093  **0.049***  -0.151  **0.001***  -0.373  **<0.000*** | 1.000  0.268  **0.000***  -0.300  **<0.000***  -0.139  **<0.003*** | 1.000  0.006  0.902  -0.012  0.803 | 1.000  0.218  **<0.000*** | 1.000 |

All variables are represented as z-scores adjusted for age, gender and pubertal status. SysBP and WC were adjusted for height. All non-BDNF blood measurements were adjusted for weekday. *and bold values = Significant correlation.
